# Evidence of a Relationship Between Plasma Leptin, Not Nesfatin-1, and Craving in Male Alcohol-Dependent Patients After Abstinence

**DOI:** 10.3389/fendo.2020.00159

**Published:** 2020-03-24

**Authors:** Ya-Yun Xu, Jin-Fang Ge, Jun Chen, Jun Liang, Liang-Jun Pang, Wen-Fan Gao, Yin Cao, Feng Shan, Yang Liu, Chun-Yu Yan, Qing-Rong Xia

**Affiliations:** ^1^Department of Pharmacy, Hefei Fourth People's Hospital, Hefei, China; ^2^CAS Key Laboratory of Brain Function and Disease, University of Science and Technology of China, Hefei, China; ^3^Psychopharmacology Research Laboratory, Anhui Mental Health Center, Hefei, China; ^4^Clinical Pharmacy, Affiliated Psychological Hospital of Anhui Medical University, Hefei, China; ^5^School of Pharmacy, Anhui Medical University, Hefei, China; ^6^Department of Material Dependence, Hefei Fourth People's Hospital, Hefei, China

**Keywords:** alcohol use disorder, brain-derived neurotrophic factor, cortisol, inflammatory cytokines, leptin, nesfatin-1

## Abstract

The goal of this study was to determine whether the plasma leptin, nesfatin-1, cortisol, brain-derived neurotrophic factor (BDNF), and inflammatory cytokines could be used as potential biomarkers for the degree of craving in the alcohol-dependent patients after 1 month of abstinence. A total of 83 patients with alcohol use disorder (AUD) and 61 healthy subjects were assessed. Patients with AUD were selected from Department of Material Dependence, Anhui Mental Health Center, and subjects in the control group were selected from healthy volunteers. The Alcohol Urge questionnaire Scale (AUQ) was used to evaluate the extent of craving for alcohol, and the Michigan Alcoholism Screening Test (MAST), the Fagerstrom Test for Nicotine Dependence (FTND), the Self-Rating Anxiety Scale (SAS), and the Self-Rating Depression Scale (SDS) were also assessed in patients with AUD. Enzyme-Linked Immunosorbent Assay (ELISA) was used for the measurement of plasma leptin, nesfatin-1, cortisol, BDNF, Interleukin-6 (IL-6), C-reactive protein (CRP), and tumor necrosis factor-α (TNF-α) levels. Compare with healthy controls, the average leptin, leptin/BMI, IL-6, CRP, and TNF-α levels in patients with AUD were significantly increased, while the BDNF levels were significantly decreased. Moreover, the partial correlational analysis showed that the AUQ scores of the alcohol-dependent patients were positively correlated with the plasma leptin levels (*r* = 0.613, *P* < 0.001), rather than nesfatin-1 (*r* = 0.066, *P* = 0.569) after controlling for age as covariate. Furthermore, plasma nesfatin-1 levels were found to be correlated with the SDS scores (*r* = 0.366, *P* = 0.001) in the AUD group. In addition, plasma leptin levels were positively associated with the plasma IL-6 (*r* = 0.257, *P* = 0.033), CRP (*r* = 0.305, *P* = 0.011), and TNF-α (*r* = 0.311, *P* = 0.009) levels, and negatively associated with the BDNF levels (*r* = −0.245, *P* = 0.042) in patients with AUD. These results suggest that plasma leptin, but not nesfatin-1, might be a potential biomarker for the degree of craving in alcohol-dependent patients after 1 month of abstinence, the mechanism of which might be related to the dysfunction of the inflammatory cytokines and BDNF levels.

## Introduction

According to the World Health Organization, 3 million people die every year from harmful use of alcohol, accounting for 5.3% of all deaths. Harmful use of alcohol is responsible for more than 200 diseases and injuries. In addition to health consequences, harmful use of alcohol can cause significant social and economic losses to individuals and society as a whole (1). There is evidence that approximately 90% of alcoholics may experience at least one relapse within 4 years after treatment (2), which may be related to the high craving. Craving, the persistent urge or desire to use a substance, has been reintroduced as a standard for defining alcohol use disorders in DSM-5, emphasizing its pivotal role in the treatment of dropouts and relapses (3). Therefore, the description of the determinants of cravings and their treatment is a key goal of alcohol use disorder (AUD) research and may constitute an innovative lever to reduce recurrence rates.

Recent evidence has shown the role of appetite-regulating peptide hormones in AUD, especially in the neurobiology of alcohol craving. A number of studies have demonstrated that serum leptin (4, 5) or ghrelin (6, 7) levels were found to be changed during alcohol craving and in chronic alcohol consumption. Moreover, a power-based analysis showed that serum leptin level was associated with alcohol craving in both sexes (8). Nesfatin-1, another new anorexigenic peptide hormone, plays an important role in the integration of food intake, energy expenditure, and glucose homeostasis (9). In the past decade, nesfatin-1 has been found to be involved in a variety of affective disorders, including anxiety disorder (10), depression (11), and psychosis (12). However, no studies on the relationship between nesfatin-1 in AD and craving have been conducted. Taken together the fact that nesfatin-1 has an appetite-regulating effect similar to that of leptin, it is rational to assume that nesfatin-1 might associate with alcohol craving in patients with AUD after abstinence.

AUD causes changes in the innate and adaptive immune response and often coincides with inflammation (13). Recent clinical study showed that inflammatory cytokines were closely linked to the craving of alcohol-related diseases. It has been reported that blood levels of IL-6 decreased during alcohol withdrawal (14), and IL-6 and TNF-α levels were associated with affective symptoms in patients with AUD. Particularly, the levels of TNF-α and IL-6 in peripheral blood were positively correlated with craving during alcohol withdrawal (15). Moreover, TNF-α release in alcohol-dependent patients was associated with the duration of abstinence (16). Therefore, whether these inflammatory cytokines could serve as objective and reliable biological indicators for the degree of craving in patients with AUD was evaluated in the present study.

Brain-derived neurotrophic factor (BDNF) and cortisol regulation are linked by a variety of mechanisms (17), and levels of each are modified under stress response, which is involved in the neurobiology of alcohol craving (18). Clinical study has shown that low serum BDNF levels are associated with characteristics related to alcohol consumption (19). Conversely, high cortisol levels have largely been associated with high alcohol consumption, which may be due to the imbalance of HPA axis (20). Therefore, whether the plasma concentrations of BDNF and cortisol were correlated with the degree of craving in patients with AUD was investigated.

Because of the dysfunction of appetites, with the important role of inflammatory markers and BDNF in the pathophysiology of AUD, the aim of the present study was to investigate whether plasma nesfatin-1, leptin, inflammatory cytokines, BDNF, and cortisol could be used as novel non-invasive biomarkers the degree of craving in patients with AUD after 1 month of abstinence.

## Materials and Methods

### Subjects

**Figure 1:**
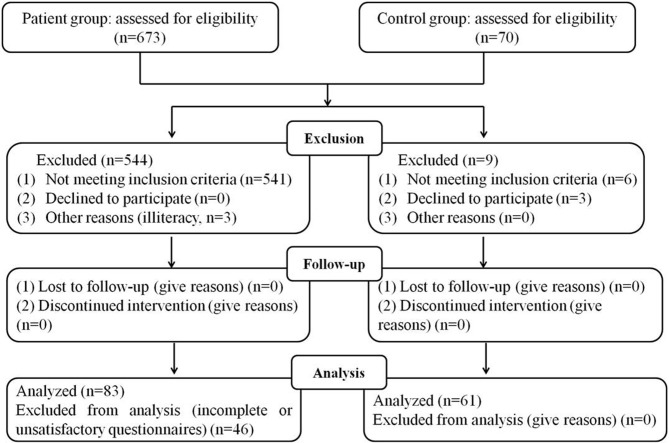
Flowchart showing the recruitment of participant.

This study was conducted at Hefei Fourth People's Hospital, Anhui Mental Health Center, between August 2017 and October 2018. A total of 673 subjects with AUD were screened by an experienced researcher in accordance with the guidelines of the structured clinical interview according to International Classification of Diseases 10th Revision (ICD-10) criteria, and 83 patients with AUD were selected from the above patients. The criteria for joining the group are as follows: (1) age 18 to 65 years; (2) completion of benzodiazepines substitution for decreasing dependence therapy; (3) Clinical Institute Withdrawal Assessment-Advanced Revised (CIWA-AR) scores < 5; (4) giving written informed consent to participate in the study. The exclusion criteria were as follows: (1) suffer from mental disorders; (2) diagnosed of substance dependent diseases other than tobacco; (3) suffering from a major neurological or medical illness. In terms of control group, 61 healthy volunteers who had not reported ethanol consumption during any period of their life were included. In order to avoid the influence of daily energy and macronutrients intakes on alcohol craving and the plasma levels of appetite-related molecules, both groups took the same food, and they were stated not to drink sugary beverage until drawing blood sample. Meals for both groups of subjects were provided by the cafeteria of Anhui Mental Health Center. Dietary intakes were assessed with a validated quantitative food frequency questionnaire by trained students via a face-to-face interview (21). The food intake of each meal was recorded for 7 consecutive days. The daily intakes of nutrients and energy, macronutrients, cholesterol and fatty acids were calculated according to the Chinese Food Composition Database (22). The recruitment process was summarized in Figure 1. In accordance with the principles of the Declaration of Helsinki, all subjects provided informed written consent prior to participation. The Ethics Committee of Hefei Fourth People's Hospital, Anhui Mental Health Center, approved this study.

### Clinical Data and Scales Measurements

Alcohol Urge Questionnaire Scale (AUQ) was adopted to assess the extent of craving at abstinence phase. The Michigan Alcoholism Screening Test (MAST) was used to identify current or potential alcohol problems among patients. The Fagerstrom Test for Nicotine Dependence (FTND) was used to evaluate the severity of nicotine dependence. The Self-Rating Anxiety Scale (SAS) and the Self-Rating Depression Scale (SDS) were used to assess the degree of anxiety and depression. A Demographics Questionnaire was used to collect general information about participants, such as years of education and sex, body weight, body-mass-index (BMI), years since first alcohol-related problems and first physical withdrawal symptoms occurred, number of inpatient detoxifications, amount of alcohol consumed daily, family history of alcoholism, and routine laboratory parameters including total bilirubin (TBIL), direct bilirubin (DBIL), alanine aminotransferase (ALT), aspartate aminotransferase (AST), alkaline phosphatase (ALP), γ-glutamyltranspeptidase (γ-GGT), total bile acid (TBA), total cholesterol (TC), high density lipoprotein (HDL), triglyceride (TG), fasting glucose, free triiodothyronine (fT3), free thyroxine (fT4), and thyroid-stimulating hormone (TSH).

### Biochemical Measurements

The blood sample was taken from the participant's vein between 8:00 a.m. and 9:00 a.m. at the end of an overnight fasting period range of 10–11 h. Tubes containing ethylenediaminetetraacetic acid (EDTA) were used for collecting the samples. The blood samples were immediately centrifuged at 3,000 rpm for 5 min at 4°C. Extract supernatant as plasma sample was collected. The extracted plasma was stored at −80°C until detection. Commercially available Enzyme-Linked Immunosorbent Assay (ELISA) kits were used to measure the concentrations of nesfatin-1, leptin, cortisol, BDNF, IL-6, CRP, and TNF-α (Jianglai Bio, Shanghai, China) according to the manufacturer's instructions. The assay sensitivities for leptin, nesfatin-1, IL-6, TNF-α, CRP, cortisol, and BDNF were 0.1 pg/ml, 0.1 pg/ml, 10 pg/ml, 1.0 pg/ml, 0.1 mg/L, 0.1 nmol/L, and 1.0 pg/ml, respectively, with an intra-assay variation of less than 9.0% and an inter-assay variation of <11.0%.

### Statistical Analysis

Data were analyzed using SPSS version 17.0 statistical analysis software (SPSS, Chicago, IL). The differences between groups were evaluated by student's *t*-test or χ^2^ test. Additional Bonferroni correction for multiple hypothesis testing considered the results statistically at a significance level of 0.05/7 = 0.007 according to the seven proteins or steroids analyzed in Table 2. Due to the large age range (18–65 years) of male tested in the present study, a partial correlational analysis was used to explore the relationship between the plasma leptin or nesfatin-1 concentrations and AUQ scores, SDS scores, inflammatory makers and BDNF levels in patients with AUD, controlling for age as covariate. Quantitative data are reported as means ± standard deviation (SD).

## Results

### Demographic Values, Alcohol-Related Data, Routine Laboratory Parameters and Scales of the AD Group and the Control Group

As shown in Table 1, there were no significant differences in age, BMI, dietary intakes, or sex between the two groups. The first drinking age of alcohol-dependent patients were average of 17.87 ± 3.24 years old, with a reported daily intake of ethanol ranging from 4 to 33 standard drink (mean 10.54 ± 6.10 standard drink, American standard) over a period of 7.56 ± 6.62 years.

**Table 1:** Comparison of demographic data, alcohol-related data, routine laboratory parameters, and scales between alcohol-dependent patients and healthy controls.

	**Control group**	**Patients' group**	***P* value**
Age	42.39 ± 11.41	39.16 ± 9.06	0.07
BMI (kg/m^2^)	23.43 ± 1.49	21.98 ± 3.47	0.195
Dietary intakes			
Energy (kcal/d)	1820 ± 306	1710 ± 314	0.438
Carbohydrate (g/d)	200.33 ± 48.50	170.00 ± 36.06	0.434
Protein (g/d)	64.67 ± 13.50	61.66 ± 18.50	0.832
Total fat (g/d)	61.01 ± 13.53	65.33 ± 14.29	0.722
Years of drinking	n.a.	7.56 ± 6.62	n.a.
Daily intake (standard drink)	n.a.	10.54 ± 6.10	n.a.
First drinking age	n.a.	17.87 ± 3.24	n.a.
TBIL (umol/L)	7.32 ± 3.59	8.55 ± 3.47	0.083
DBIL (umol/L)	3.24 ± 1.16	3.13 ± 1.43	0.678
ALT (U/L)	15.03 ± 7.64	34.75 ± 23.41	<0.001^**^
AST (U/L)	17.19 ± 4.89	28.12 ± 13.85	<0.001^**^
ALP (U/L)	63.86 ± 18.19	80.19 ± 24.41	<0.001^**^
γ-GGT (U/L)	15.11 ± 5.66	107.16 ± 89.77	<0.001^**^
TBA (umol/L)	3.72 ± 2.54	7.14 ± 6.75	0.004^**^
TC (umol/L)	4.10 ± 0.59	4.97 ± 1.11	<0.001^**^
HDL (mmol/L)	1.19 ± 0.18	1.19 ± 0.27	0.887
TG (mmol/L)	1.21 ± 0.52	1.73 ± 0.75	<0.001^**^
fT3 (pg/ml)	2.62 ± 0.38	2.71 ± 0.49	0.323
fT4 (ng/dl)	1.02 ± 0.18	0.92 ± 0.18	0.005^**^
TSH (ng/ml)	1.63 ± 1.11	1.94 ± 2.03	0.398
Fasting glucose (mmol/L)	4.67 ± 0.48	4.57 ± 0.68	0.423
AUQ	n.a.	19.53 ± 8.39	n.a.
MAST	n.a.	30.47 ± 9.44	n.a.
FTND	n.a.	5.14 ± 2.01	n.a.
SAS	n.a.	43.45 ± 8.51	n.a.
SDS	n.a.	48.17 ± 11.25	n.a.

*BMI, body-mass-index; TBIL, total bilirubin; DBIL, direct bilirubin; ALT, alanineaminotransferase; AST, aspartate aminotransferase; ALP, alkaline phosphatase; γ-GGT, γ-glutamyltranspeptidase; TBA, total bile acid; TC, total cholesterol; HDL, high density lipoprotein; TG, triglyceride; fT3, free triiodothyronine; fT4, free thyroxine; TSH, thyroid-stimulating hormone; AUQ, Alcohol Urge Questionnaire Scale; MAST, Michigan Alcoholism Screening Test; FTND, Fagerstrom Test for Nicotine Dependence; SAS, Self-Rating Anxiety Scale; SDS, Self-Rating Depression Scale*.

** *P < 0.01 was considered statistically significant*.

The mean ALT, AST, ALP, γ-GGT, and TBA levels were significantly higher in the AUD group when compared with the control group, which suggested that liver function of the AUD patients has not fully recovered after a month of abstinence. Moreover, the mean TC and TG levels of the AUD patients were also increased statistically, indicating that there was still a disorder of lipid metabolism in the patients with AUD receiving a month of abstinence.

The average scores of AUQ, MAST, FTND, SAS, and SDS of alcohol-dependent patients were 19.53 ± 8.39, 30.47 ± 9.44, 5.14 ± 2.01, 43.45 ± 8.51, and 48.17 ± 11.25, respectively (Table 1).

### Circulating Inflammatory Cytokines, Leptin, Nesfatin-1, Cortisol, and BDNF Levels of the AUD Group and the Control Group

In order to control for interactions of BMI with leptin or nesfatin-1 levels, the leptin/BMI ratio and nesfatin-1/BMI ratio were also calculated and analyzed between the two groups. As shown in Table 2, the mean leptin levels (*t* = −9.375, *P* < 0.001) and leptin/BMI ratio (*t* = −7.990, *P* < 0.001) were significantly higher, while the mean BDNF levels (*t* = 2.929, *P* = 0.008) were significantly lower in the AUD group than in the control group. In terms of inflammatory cytokines, the plasma concentrations of IL-6 (*t* = −2.339, *P* = 0.021), CRP (*t* = −4.990, *P* < 0.001), and TNF-α levels (*t* = −7.969, *P* < 0.001) in the AUD group were increased significantly than those in the healthy control group. No statistical difference was observed in nesfatin-1 levels (*t* = 1.256, *P* = 0.211), nesfatin-1/BMI ratio (*t* = 1.145, *P* = 0.255), and cortisol (*t* = 0.985, *P* = 0.326) between the two groups. However, only the mean leptin, CRP, and TNF-α levels remained significance after Bonferroni correction.

**Table 2:** Comparison of leptin, leptin/BMI, nesfatin-1, nesfatin-1/BMI, inflammatory cytokines, cortisol, and BDNF levels between alcohol-dependent patients and healthy controls after 1 month of abstinence.

	**Control group**	**Patients' group**	***t***	***P* value**
Leptin (ng/ml)	3.05 ± 1.16	5.42 ± 1.69	−9.375	<0.001^a^
Leptin/BMI	0.13 ± 0.03	0.25 ± 0.08	−7.990	<0.001^a^
Nesfatin-1 (ng/ml)	1.56 ± 0.30	1.50 ± 0.31	1.256	0.211
Nesfatin-1/BMI	0.066 ± 0.015	0.069 ± 0.013	1.145	0.255
IL-6 (pg/ml)	34.01 ± 13.73	39.38 ± 13.33	−2.339	0.021^*^
TNF-α (pg/ml)	46.52 ± 20.67	75.61 ± 22.07	−7.969	<0.001^**^^a^
CRP (mg/L)	8.37 ± 3.00	11.24 ± 3.64	−4.990	<0.001^**^^a^
Cortisol (nmol/L)	214.97 ± 60.05	204.78 ± 61.50	0.985	0.326
BDNF (ng/ml)	57.06 ± 21.43	48.66 ± 12.33	2.929	0.008^**^

*IL-6, interleukin-6; CRP, C-reactive protein; TNF-α, tumor necrosis factor-α; BDNF, Brain-derived neurotrophic factor*.

* *P < 0.05*,

** *P < 0.01 was considered statistically significant*.

a *Statistically significant even after additional Bonferroni correction for multiplicity*.

### Relationship Between Leptin or Nesfatin-1 Levels and Craving in Patients With AUD

The results of the partial correlational analysis showed that the AUQ scores were positively correlated with plasma leptin concentrations (*r* = 0.613, *P* < 0.001; Table 3), but not plasma nesfatin-1 concentrations (*r* = 0.066, *P* = 0.569; Table 3) in patients with AUD after controlling for age as covariate.

**Table 3:** Partial correlation between the plasma leptin or nesfatin-1 concentrations and AUQ scores, SDS scores, inflammatory makers and BDNF levels in patients with AUD after controlling for age.

	**Leptin**		**Nesfatin-1**	
	***r***	***P* value**	***r***	***P* value**
AUQ	0.613	<0.001^**^	0.066	0.569
SDS	−0.066	0.564	0.366	0.001^**^
IL-6	0.257	0.033^*^	−0.094	0.412
TNF-α	0.311	0.009^**^	−0.056	0.628
CRP	0.305	0.011^*^	0.112	0.329
BDNF	−0.245	0.042^*^	−0.056	0.625

*AUQ, Alcohol Urge Questionnaire Scale; SDS, Self-Rating Depression Scale; IL-6, interleukin-6; CRP, C-reactive protein; TNF-α, tumor necrosis factor-α; BDNF, Brain-derived neurotrophic factor*.

* *P < 0.05*,

** *P < 0.01 was considered statistically significant*.

### Relationship Between Leptin or Nesfatin-1 Levels and Degree of Depression in Patients With AUD

As shown in Table 3, plasma nesfatin-1 concentrations were positively correlated with the SDS scores (*r* = 0.366, *P* = 0.001) in the alcohol-dependent patients after controlling for age as covariate. However, no correlation between SDS scores and leptin was observed in patients with AUD (*r* = −0.066, *P* = 0.564; Table 3).

### Relationship Between Leptin or Nesfatin-1 Levels and Inflammatory Makers and BDNF Levels in Patients With AUD

As shown in Table 3, plasma leptin concentrations were positively correlated with the plasma IL-6 (*r* = 0.257, *P* = 0.033), CRP (*r* = 0.305, *P* = 0.011), and TNF-α (*r* = 0.311, *P* = 0.009), whereas negative relationship was found between leptin levels and BDNF levels (*r* = −0.245, *P* = 0.042; Table 3) in the AUD group after controlling for age as covariate. Moreover, plasma nesfatin-1 concentrations were not correlated with the plasma IL-6 (*r* = −0.094, *P* = 0.412), CRP (*r* = 0.112, *P* = 0.329), TNF-α (*r* = −0.056, *P* = 0.628), and BDNF levels (*r* = −0.056, *P* = 0.625; Table 3) in the alcohol-dependent patients.

Additionally, the plasma leptin concentrations were not correlated with the ALT, AST, ALP, γ-GGT, TBA, TC, and TG levels (*P* > 0.05) in the alcohol-dependent patients.

## Discussion

In the present study, we demonstrated that the average leptin, IL-6, CRP, and TNF-α levels were significantly higher in the AUD patients when compared with healthy controls. Moreover, the partial correlational analysis showed that the AUQ scores were positively correlated with the plasma leptin levels in patients with AUD. Furthermore, the plasma nesfatin-1 levels were found to be correlated with the SDS scores, but not the AUQ scores in alcohol-dependent patients. Additionally, the plasma leptin levels were positively associated with the plasma IL-6, CRP, and TNF-α levels, and negatively associated with the BDNF levels in patients with AUD. These results suggest that the plasma leptin, but not nesfatin-1, might be a potential biomarker for the degree of addiction in alcohol-dependent patients after 1 month of abstinence, and its mechanism may be related to inflammatory dysfunction and BDNF levels.

Patients with AUD in this study underwent alcohol dependence rehabilitation treatment program for 1 month that includes oral administration of benzodiazepines during the abstinence period for protracted alcohol-withdrawal symptoms or behavior control. After that, the patient has basically reached discharge standards. Thus, one month after withdrawal was chosen as a time point to evaluate the degree of craving and find possible biomarkers.

The issue of appetite peptide hormones as putative state markers of alcohol use and its role in craving has been raised in the last few years. Clinical studies have indicated that increased levels of leptin were significantly associated with the amount of alcohol consumed by active drinkers throughout their lives, regardless of nutritional status or the presence of compensated liver disease (23). Preclinical studies showed that chronic ethanol consumption increased circulating levels of leptin with a change in expression of leptin sensing molecules in the hypothalamus and peripheral adipose tissue (24). In line with these results, in the present study, the patients with AUD after alcohol abstinence showed higher plasma leptin levels compared with healthy control subjects. Recently, an association between leptin and craving in females has been observed (25). In this study, patients with AUD were all male. Based on the partial correlational analysis, our results showed that the plasma leptin levels were associated with the AUQ scores in male alcohol-dependent patients after alcohol abstinence. These results indicated that plasma leptin might be a potential biomarker for the degree of addiction in both sexes.

Alcohol-dependent patients often have liver and metabolic disorders. In the present study, the AUD group exhibited significantly increased ALT, AST, ALP, γ-GGT, and TBA levels, and TC and TG levels when compared with healthy controls, which suggested that liver function and lipid metabolism of the AUD patients has not fully recovered after a month of abstinence, respectively. Conflicting results have been reported regarding the association of plasma leptin with metabolic alterations and liver function in several diseases. It has been reported that serum leptin was clearly correlated with markers of metabolism and liver function in critically ill patients (26). By contrast, in another study, no association of routine laboratory parameters, especially liver enzymes (gGT, AST, ALT) and leptin levels was observed in alcohol addicts during an inpatient detoxification (27). Consistently, in the present study, plasma leptin concentrations were not correlated with the markers of lipid metabolism and liver function further suggesting that serum leptin not correlated with the abnormal lipid metabolism and liver function in the alcohol-dependent patients after abstinence.

Ghrelin, the counter hormone of leptin, has been reported to play an important role in the neurobiology of AUD. Clinical studies have shown a correlation between ghrelin levels and alcohol craving in alcoholics (28). Specifically, baseline ghrelin levels (Ghrelin-T0) were significantly and positively correlated with the craving scores in alcohol-dependent subjects, but Ghrelin-T1 (2 weeks after baseline), Ghrelin-T2 (6 weeks) and Ghrelin-T3 (12 weeks) did not correlate with any of the alcohol craving scores (28). These results indicated that ghrelin levels at baseline, not after withdrawal, were associated with alcohol craving in alcoholics. Given the fact that the aim of the present study was to investigate the relationship between plasma biomarkers and the degree of craving in patients with AUD after 1 month of abstinence, the levels of ghrelin were not collected.

Nesfatin-1, another anorexigenic hormone, has a variety of neuroendocrine functions, including emotional regulation, stress response, inflammatory response, cardiovascular system and reproduction (29). Few studies on the correlation between nesfatin-1 and alcohol dependence has been reported. Only one study showed that plasma NUCB2/nesfatin-1 levels were lower in highest craving phase and tended to normalize after abstinence (30). Consistently, no statistical difference was observed in nesfatin-1 levels between the alcohol-dependent patients and healthy controls, and neither was the correlation between craving and nesfatin-1 levels, indicating that nesfatin-1 was not suitable as a marker of severity of addiction in patients with AUD after withdrawal. Recent studies have suggested that there is a close relationship between nesfatin-1 and depression (31). Our previous results (32, 33) together with other studies (34) have demonstrated that the plasma nesfatin-1 level was positively correlated with the severity of depression. In the present study, SDS, a 20-item self-report questionnaire that is widely used as a screening tool, covering affective, psychological, and somatic symptoms associated with depression, was used to evaluate the severity of depression in patients with AUD after withdrawal. The results showed that the plasma nesfatin-1 concentrations were positively correlated with the SDS scores, providing more data linking nesfatin-1 to depression.

Accumulated data support a certain link between AUD and inflammatory response. It has been indicated that regardless of the amount of alcohol consumed, the serum TNF-α concentration in alcoholic patients was higher than that in the general population (35). Moreover, IL-6 serum levels were directly associated with alcohol consumption on the first day of withdrawal (35). In addition, the levels of IL-6, IL-12, and TNF-α synthesis were significantly increased among chronic alcoholics still consuming alcohol (36). Consistently, the present study showed that the plasma levels of IL-6, CRP, and TNF-α were significantly increased in the patients with AUD, which again suggests that inflammatory factors may play an important role in AUD. Leptin, an adipocyte-secreted hormone that centrally regulates weight control, is the cornerstone of regulating immune and inflammatory response (37, 38). Previous studies have indicated that serum TNF-α levels were positively correlated with leptin levels in patients with systemic sclerosis (39). Another study showed that leptin could induce IL-6 expression through the long form (OBRl) leptin receptor (40). Plasma leptin concentrations were positively correlated with the plasma IL-6, CRP, and TNF-α levels in the present study, which suggests that the role of leptin as a marker of addiction in alcohol-dependent patients may be related to the changes in inflammatory cytokines.

Recent studies have hypothesized that prolonged alcohol intake may affect the synthesis of BDNF, which has a significant role in neuronal development, plasticity and learning. It has been reported that serum BDNF levels were significantly lower in alcoholic patients compared with healthy controls (19). Another study found that the level of BDNF in the seventh day of withdrawal was lower than that of alcohol dependent patients before abstinence (41). Recently, a significant association of BDNF with alcohol craving measured by the Obsessive and Compulsive Drinking Scale (OCDS) was been observed (42). In consonance with these data, the levels of BDNF in the AUD group were lower than that in control group in the present study. However, the correlation between BDNF and alcohol dependence was not observed, which might due to the choice of scale or the different withdrawal period. Additionally, a negative relationship between leptin and BDNF levels was observed. Taken together the fact that hypothalamic BDNF down-regulated leptin production in adipocytes via sympathoneural β-adrenergic signaling (43), it is rational to presume that the role of leptin as an addictive marker in alcohol-dependent patients may be related to the levels of BDNF.

Recent study explored the effects of alcohol on cortisol levels. It has been revealed that the cortisol concentrations of alcoholics were three to four times higher than those of alcoholics or non-alcoholics (44). However, abstinent alcoholics and non-alcoholics showed similar low cortisol levels (44), indicating that cortisol levels eventually returned to normal after long-term alcohol withdrawal. In accordance with these results, present study showed that after a month of abstinence, similar cortisol levels were observed in the alcohol-dependent patients and healthy controls, indicating that plasma cortisol might not suitable as a biomarker for alcohol-dependent addiction in alcohol-dependent patients after withdraw.

The basal level of data in alcohol-dependent patients and controls before abstinence should also be included for the reason that we can dynamically observe the correlation between the plasma leptin levels and craving. However, almost all the patients in the present study do not have the ability or refuse to sign informed consent based on their state at the time of admission. Thus, these data at baseline were not collected. Due to fact that ~90% of alcoholics may experience at least one relapse within 4 years after treatment (1), it is of great clinical significance to the description of the determinants of cravings after treatment and discharge in alcohol-dependent patients and to reduce the recurrence rate. Therefore, the data of patients after abstinence and treatment were collected, and the aim of the present study was to find possible biomarkers for the degree of craving in alcohol-dependent patients after alcohol dependence rehabilitation treatment program for 1 month. Based on the current data, we may draw the conclusion that plasma leptin might be a potential biomarker for the degree of craving in alcohol-dependent patients after 1 month of abstinence. Multi-centric and longitudinal studies are clearly required to dynamically investigate whether plasma leptin could be used as novel non-invasive biomarkers the degree of craving in patients with AUD.

This study has several limitations. Firstly, this study is a single-center study with relatively small sample size. Secondly, the patients with AUD in this study were all male, which might be considered as a limitation. Thirdly, only one time point after withdrawal was observed, and the data at baseline were not included. Further studies are needed to dynamically observe and identify biomarkers of AUD and addiction.

At the end of this study, we have reached upon this conclusion that plasma leptin, rather than nesfatin-1, might be a potential biomarker for alcohol-dependent addiction in alcohol-dependent patients after 1 month of abstinence, and its mechanism may be related to abnormalities in inflammatory factors and BDNF levels. The exact mechanism needs further study to explore.

## Data Availability Statement

The raw data supporting the conclusions of this article will be made available by the authors, without undue reservation, to any qualified researcher.

## Ethics Statement

The studies involving human participants were reviewed and approved by The Ethics Committee of Hefei Fourth People's Hospital, Anhui Mental Health Center. The patients/participants provided their written informed consent to participate in this study.

## Author Contributions

J-FG, Q-RX, and Y-YX designed the study, wrote the protocol, and the first draft of the manuscript. Y-YX managed the literature searches and the statistical analyses. Y-YX, JC, JL, L-JP, W-FG, YC, FS, YL, and C-YY performed the experiments. All authors contributed to and have approved the final manuscript.

### Conflict of Interest

The authors declare that the research was conducted in the absence of any commercial or financial relationships that could be construed as a potential conflict of interest.

## References

[1] WHO Alcohol. Available online at: http://www.who.int/mediacentre/factsheets/fs349/en/index.html (Last accessed December 17, 2018).

[2] Barnett R. Alcohol use disorders. Lancet. (2017) 389:25. 10.1016/S0140-6736(16)32600-9 28093128

[3] Hartwell EE Ray LA. Craving as a DSM-5 symptom of alcohol use disorder in non-Treatment seekers. Alcohol. (2018) 53:235–40. 10.1093/alcalc/agx088 29145640

[4] Proskynitopoulos PJ Rhein M Jackel E Manns MP Frieling H Bleich S Leptin expression and gene methylation patterns in alcohol-dependent patients with ethyltoxic cirrhosis-normalization after liver transplantation and implications for future research. Alcohol. (2018) 53:511–7. 10.1093/alcalc/agy038 29912265

[5] Geisel O Hellweg R Wiedemann K Muller CA. Plasma levels of leptin in patients with pathological gambling, internet gaming disorder and alcohol use disorder. Psychiatry Res. (2018) 268:193–7. 10.1016/j.psychres.2018.06.042 30041134

[6] Koopmann A Lippmann K Schuster R Reinhard I Bach P Weil G. Drinking water to reduce alcohol craving? A randomized controlled study on the impact of ghrelin in mediating the effects of forced water intake in alcohol addiction. Psychoneuroendocrinology. (2017) 85:56–62. 10.1016/j.psyneuen.2017.08.005 28822300

[7] Morris LS Voon V Leggio L Stress, motivation, and the gut-brain axis: a focus on the ghrelin system and alcohol use disorder. Alcohol Clin Exp Res. (2018) 42:1378–89. 10.1111/acer.13781 PMC6252147 29797564

[8] Hillemacher T Bleich S Frieling H Schanze A Wilhelm J Sperling W. Evidence of an association of leptin serum levels and craving in alcohol dependence. Psychoneuroendocrinology. (2007) 32:87–90. 10.1016/j.psyneuen.2006.09.013 17095166

[9] Schalla MA Stengel A. Current understanding of the role of nesfatin-1. J Endocr Soc. (2018) 2:1188–206. 10.1210/js.2018-00246 30302423 PMC6169466

[10] Ge JF Xu YY Qin G Pan XY Cheng JQ Chen FH. Nesfatin-1, a potent anorexic agent, decreases exploration and induces anxiety-like behavior in rats without altering learning or memory. Brain Res. (2015) 1629:171–81. 10.1016/j.brainres.2015.10.027 26498879

[11] Algul S Ozcelik O. Evaluating the levels of nesfatin-1 and ghrelin hormones in patients with moderate and severe major depressive disorders. Psychiatry Investig. (2018) 15:214–8. 10.30773/pi.2017.05.24 29475222 PMC5900400

[12] Sahpolat M Ari M. Plasma nesfatin 1 level in patients with first attack psychosis. Bratisl Lek Listy. (2017) 118:77–9. 10.4149/BLL_2017_015 28814086

[13] Leclercq S de Timary P Delzenne NM Starkel P. The link between inflammation, bugs, the intestine and the brain in alcohol dependence. Transl Psychiatry. (2017) 7:e1048. 10.1038/tp.2017.15 28244981 PMC5545644

[14] Zgierska A Rabago D Zuelsdorff M Coe C Miller M Fleming M. Mindfulness meditation for alcohol relapse prevention: a feasibility pilot study. J Addict Med. (2008) 2:165–73. 10.1097/ADM.0b013e31816f8546 21768988 PMC4106278

[15] Leclercq S Cani PD Neyrinck AM Starkel P Jamar F Mikolajczak M. Role of intestinal permeability and inflammation in the biological and behavioral control of alcohol-dependent subjects. Brain Behav Immun. (2012) 26:911–8. 10.1016/j.bbi.2012.04.001 22521198

[16] Kiefer F Jahn H Schick M Wiedemann K. Alcohol intake, tumour necrosis factor-alpha, leptin and craving: factors of a possibly vicious circle? Alcohol Alcohol. (2002) 37:401–4. 10.1093/alcalc/37.4.401 12107045

[17] Munoz MJ Kumar RG Oh BM Conley YP Wang Z Failla MD. Cerebrospinal fluid cortisol mediates brain-derived neurotrophic factor relationships to mortality after severe TBI: a prospective cohort study. Front Mol Neurosci. (2017) 10:44. 10.3389/fnmol.2017.00044 28337122 PMC5343043

[18] McCaul ME Hutton HE Stephens MA Xu X Wand GS. Anxiety, anxiety sensitivity, and perceived stress as predictors of recent drinking, alcohol craving, and social stress response in heavy drinkers. Alcohol Clin Exp Re. (2017) 41:836–45. 10.1111/acer.13350 28281290 PMC5388456

[19] Nubukpo P Ramoz N Girard M Malauzat D Gorwood P. Determinants of blood brain-Derived neurotrophic factor blood levels in patients with alcohol use disorder. Alcohol Clin Exp Res. (2017) 41:1280–7. 10.1111/acer.13414 28485899

[20] Dai X Thavundayil J Santella S Gianoulakis C. Response of the hPA-axis to alcohol and stress as a function of alcohol dependence and family history of alcoholism. Psychoneuroendocrinology. (2007) 32:293–305. 10.1016/j.psyneuen.2007.01.004 17349749

[21] Zhang CX Ho SC. Validity and reproducibility of a food frequency questionnaire among Chinese women in Guangdong province. Asia Pac J Clin Nutr. (2009) 18:240–50. 19713184

[22] Yang Y He M Pan X China Food Composition Book. Beijing: Peking University Medical Press (2009).

[23] Nicolas JM Fernandez-Sola J Fatjo F Casamitjana R Bataller R Sacanella E. Increased circulating leptin levels in chronic alcoholism. Alcohol Clin Exp Res. (2001) 25:83–8. 10.1111/j.1530-0277.2001.tb02130.x 11198718

[24] Obradovic T Meadows GG. Chronic ethanol consumption increases plasma leptin levels and alters leptin receptors in the hypothalamus and the perigonadal fat of c57BL/6 mice. Alcohol Clin Exp Res. (2002) 26:255–62. 10.1111/j.1530-0277.2002.tb02532.x 11964566

[25] Kraus T Reulbach U Bayerlein K Mugele B Hillemacher T Sperling W. Leptin is associated with craving in females with alcoholism. Addict Biol. (2004) 9:213–9. 10.1080/13556210412331292541 15511715

[26] Koch A Weiskirchen R Zimmermann HW Sanson E Trautwein C Tacke F. Relevance of serum leptin and leptin-receptor concentrations in critically ill patients. Mediators Inflamm. (2010) 10.1155/2010/473540 20871818 PMC2943118

[27] Kiefer F Jahn H Jaschinski M Holzbach R Wolf K Naber D. Leptin: a modulator of alcohol craving? Biol Psychiatry. (2001) 49:782–7. 10.1016/s0006-3223(01)01081-2 11331086

[28] Leggio L Ferrulli A Cardone S Nesci A Miceli A Malandrino N. Ghrelin system in alcohol-dependent subjects: role of plasma ghrelin levels in alcohol drinking and craving. Addict Biol. (2012) 17:452–64. 10.1111/j.1369-1600.2010.00308.x 21392177 PMC4974482

[29] Dore R Levata L Lehnert H Schulz C. Nesfatin-1: functions and physiology of a novel regulatory peptide. J Endocrinol. (2017) 232:R45–R65. 10.1530/JOE-16-0361 27754932

[30] Umut G Evren C Cansiz A Akkus M Karamustafalioglu N. Serum nUCB2/nesfatin-1 levels in different stages of alcohol dependence: is there a relationship with craving? Indian J Psychiatry. (2017) 59:94–9. 10.4103/psychiatry.IndianJPsychiatry_354_16 28529367 PMC5419020

[31] Weibert E Hofmann T Stengel A. Role of nesfatin-1 in anxiety, depression and the response to stress. Psychoneuroendocrinology. (2018) 100:58–66. 10.1016/j.psyneuen.2018.09.037 30292960

[32] Xia QR Liang J Cao Y Shan F Liu Y Xu YY. Increased plasma nesfatin-1 levels may be associated with corticosterone, iL-6, and cRP levels in patients with major depressive disorder. Clin Chim Acta. (2018) 480:107–11. 10.1016/j.cca.2018.02.004 29427582

[33] Xu YY Ge JF Liang J Cao Y Shan F Liu Y. Nesfatin-1 and cortisol: potential novel diagnostic biomarkers in moderate and severe depressive disorder. Psychol Res Behav Manag. (2018) 11:495–502. 10.2147/PRBM.S183126 30425596 PMC6202039

[34] Xiao MM Li JB Jiang LL Shao H Wang BL. Plasma nesfatin-1 level is associated with severity of depression in chinese depressive patients. BMC Psychiatry. (2018) 18:88. 10.1186/s12888-018-1672-4 29615007 PMC5883589

[35] Heberlein A Kaser M Lichtinghagen R Rhein M Lenz B Kornhuber J. TNF-alpha and iL-6 serum levels: neurobiological markers of alcohol consumption in alcohol-dependent patients? Alcohol. (2014) 48:671–6. 10.1016/j.alcohol.2014.08.003 25262503

[36] Laso FJ Vaquero JM Almeida J Marcos M Orfao A. Chronic alcohol consumption is associated with changes in the distribution, immunophenotype, and the inflammatory cytokine secretion profile of circulating dendritic cells. Alcohol Clin Exp Res. (2007) 31:846–54. 10.1111/j.1530-0277.2007.00377.x 17386065

[37] La Cava A. Leptin in inflammation and autoimmunity. Cytokine. (2017) 98:51–8. 10.1016/j.cyto.2016.10.011 27916613 PMC5453851

[38] Matarese G Leiter EH La Cava A. Leptin in autoimmunity: many questions, some answers. Tissue Antigens. (2007) 70:87–95. 10.1111/j.1399-0039.2007.00886.x 17610413

[39] Pehlivan Y Onat AM Ceylan N Turkbeyler IH Buyukhatipoglu H Comez G. Serum leptin, resistin and tNF-alpha levels in patients with systemic sclerosis: the role of adipokines in scleroderma. Int J Rheum Dis. (2012) 15:374–9. 10.1111/j.1756-185X.2012.01755.x 22898217

[40] Yang WH Liu SC Tsai CH Fong YC Wang SJ Chang YS. Leptin induces iL-6 expression through oBRl receptor signaling pathway in human synovial fibroblasts. PLoS ONE. (2013) 8:e75551. 10.1371/journal.pone.0075551 24086566 PMC3785513

[41] Cavus SY Dilbaz N Darcin AE Eren F Kaya H Kaya O Alterations in serum bDNF levels in early alcohol withdrawal and comparison with healthy controls. Bull Clin Psychopharmacol. (2012) 22:75–8. 10.1007/978-1-4471-2751-2_1

[42] Heberlein A Lenz B Opfermann B Groschl M Janke E Stange K. Association of testosterone and bDNF serum levels with craving during alcohol withdrawal. Alcohol. (2016) 54:67–72. 10.1016/j.alcohol.2016.06.004 27514572

[43] Cao L Liu X Lin EJ Wang C Choi EY Riban V. Environmental and genetic activation of a brain-adipocyte bDNF/leptin axis causes cancer remission and inhibition. Cell. (2010) 142:52–64. 10.1016/j.cell.2010.05.029 20603014 PMC3784009

[44] Stalder T Kirschbaum C Heinze K Steudte S Foley P Tietze A. Use of hair cortisol analysis to detect hypercortisolism during active drinking phases in alcohol-dependent individuals. Biol Psychol. (2010) 85:357–60. 10.1016/j.biopsycho.2010.08.005 20727937

